# Pharmaceutical compounds in marine ecosystems: ecotoxicological effects and risk assessment in phytoplankton and zooplankton

**DOI:** 10.1007/s10646-025-02960-2

**Published:** 2025-09-12

**Authors:** Roberta Nugnes, Chiara Gambardella, Roberta Miroglio, Lisa Zanetti, Marco Faimali, Francesca Garaventa

**Affiliations:** National Research Council, Institute of the Anthropic Impact and Sustainability in the marine environment (CNR-IAS), via de Marini 16, Genova, 16149 Italy

**Keywords:** Algae, Crustacean, Ecotoxicity, Marine pollution, Risk assessment, Sea urchin

## Abstract

Pharmaceutical compounds (PCs) are one of the main classes of emerging contaminants, raising concern for their potential threat to the environment. Among them, acyclovir (ACV), carbamazepine (CBZ), diclofenac (DCF), and propranolol (PRP) are the most prescribed worldwide. However, their effects on the marine environment are still little known. In this study we assessed the ecotoxicological effects of these PCs in marine phyto- and zooplankton. In particular, growth and chlorophyll inhibition, mortality, development and behaviour were assessed in several species (*Dunaliella tertiolecta*,* Phaeodactylum tricornutum*,* Skeletonema pseudocostatum*, *Amphibalanus amphitrite*,* Paracentrotus lividus*) after up to 72 h of exposure. Only high concentrations of any of the four PCs induced toxic effects at environmental levels. PRP was the most toxic PC towards both phyto- and zooplankton, followed by DCF, ACV, and CBZ. Environmental risk for all PCs could be estimated based on ecotoxicological responses, ranging from medium for ACV and CBZ, to high for DCF and PRP. In conclusion, all selected PCs may pose risk to marine phyto- and zooplankton. Hence, their presence and levels in the marine ecosystem should be further monitored.

**Graphical abstract:**

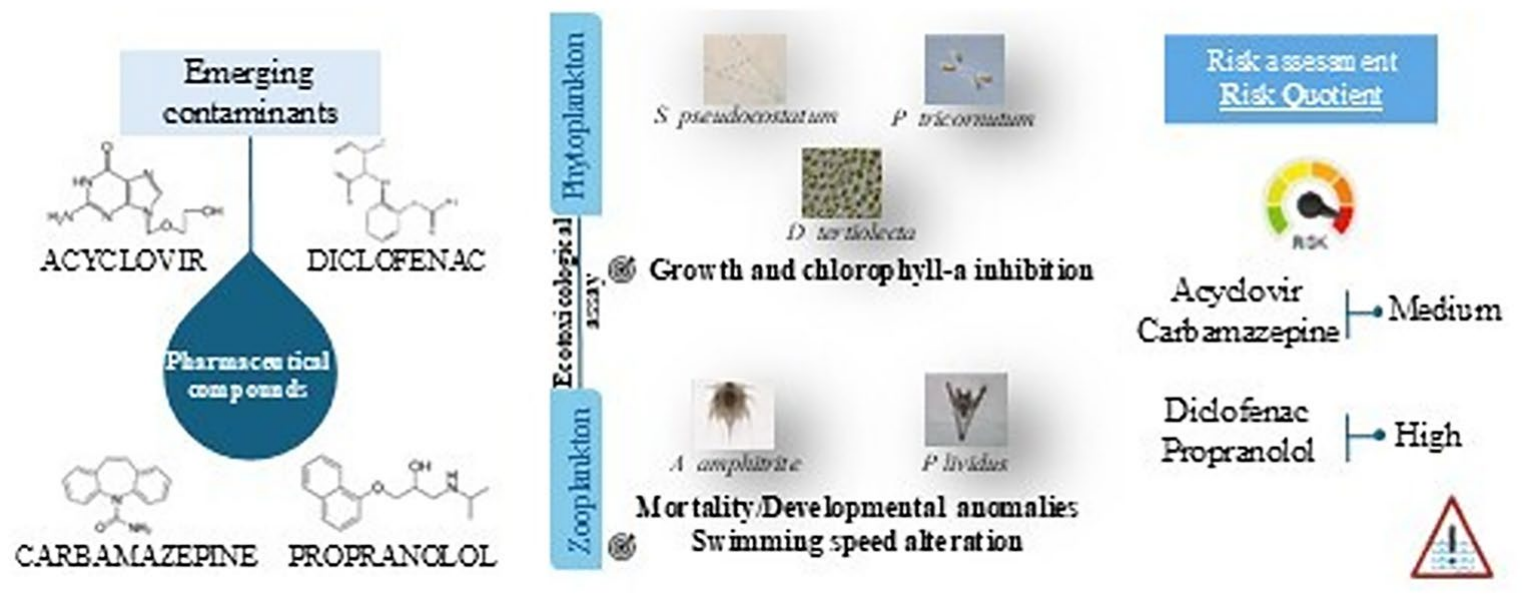

**Supplementary Information:**

The online version contains supplementary material available at 10.1007/s10646-025-02960-2.

## Introduction

Population growth, climate change, industrialization, and large-scale exploitation of natural resources have impacted on the quality of human life and environmental health. In this scenario, environmental pollution has worsened due to the widespread and massive use of some substances – such as personal care products, pesticides, nanomaterials, microplastics, and pharmaceutical compounds, defined as emerging contaminants (Puri et al. 2023). These organic compounds are found in the environment; for some of them the ecotoxicological effects are known (Puri et al. 2023; Radwan et al. 2023), while for others there is a still a lack of knowledge (Vasilachi et al. 2021). In addition, their presence is not yet routinely monitored nor adequately regulated (Rodriguez-Narvaez et al. 2017; Coccia & Bontempi 2023). Among these substances, pharmaceutical compounds (PCs) are of greatest concern due to their design for prolonged bioactivity at low concentrations. Their stable chemical structure and functional groups contribute to persistence in the environment and bioactivity, which in turn could lead to biological toxicity in both target and non-target species (Madadian & Simakov 2022). This feature makes PCs particularly relevant among the different classes of emerging contaminants to be investigated for their potential toxicity.

The global consumption of PCs has been estimated to be around 100 thousand tons per year (Gopal et al. 2020), attributable to the 12,500 therapeutic substances available on the market (Chaves et al. 2022). They belong to different classes, including antidiabetics, antihypertensives, antivirals, antibiotics, antihistamines, analgesics, anti-inflammatory and anticancer drugs, anticonvulsants, anxiolytics, antidepressants, and β-blockers. After consumption, PCs can end up in the sea water, due to inefficient wastewater treatment plants unable to fully remove them. Despite the complexity of the aquatic environment and its dilution effect (Gaw et al. 2014; Fang et al. 2019; Li et al. 2020; Madikizela et al. 2020), PCs are present worldwide in the marine environment at concentrations in the order of ng-µg/L, depending on geographic areas (Arpin-Pont et al. 2016; Mezzelani et al. 2018; Danner et al. 2019; Bhagat and Kumar 2023; Castaño-Ortiz at al. 2023). Following the Covid-19 pandemic, a progressive increase in the use of PCs and their concentration in surface waters have been recorded (Morales-Paredes et al. 2022).

PCs are considered ‘pseudo-persistent compounds’ because of their continuous release into the environment, raising concerns for marine biota. Therefore, increasing knowledge about their effects on aquatic organisms is necessary (Madikizela & Ncube 2022). While pharmaceutical toxicity has been extensively studied in freshwater species, research on marine biota is more limited, highlighting the need for further investigations (Mezzelani et al. 2018). Nowadays, few studies report molecular, cellular, and biochemical effects that could impair growth, locomotion, feeding, reproduction, and metabolism in the first levels of the marine trophic chain, namely phyto- and zooplankton (Zanuri et al. 2017; Branchet et al. 2021; Kock et al. 2023). Regarding primary producers, the exposure to anti-inflammatory, antihypertensive and antiepileptic compounds has inhibited growth, altered photosynthetic activity and induced oxidative stress in marine green algae (*Dunaliella tertiolecta*), and diatoms (*Phaeodactylum tricornutum;* De Lorenzo & Fleming 2008; Tsiaka et al. 2013; Duarte et al. 2020; Silva et al. 2020). Regarding zooplankton, crustaceans (*Tigriopus fulvus)* and sea urchin (*Paracentrotus lividus*) larvae were affected by antihypertensive drugs (i.e. propranolol) and antiepileptics (i.e. carbamazepine) in terms of reproduction and fertilization rates, respectively (Capolupo et al. 2018; Prato et al. 2023).

To date, specific regulations on PCs are not available; however, some PCs are included in the Watch List of the Water Framework Directive (WFD) 2000/60/EC (European Commission 2000). Despite the fact that the WFD consultative tool does not establish threshold limits, it does provide a list of substances to monitor for risk assessment and support policy makers. In this regard, it is paramount to include other PCs in compliance with the Marine Strategy Framework Directive (Marine Strategy Framework Directive 2008/56/EC).

Among PCs, antiviral acyclovir (ACV), antiepileptic carbamazepine (CBZ), nonsteroidal anti-inflammatory diclofenac (DCF) and antihypertensive propranolol (PRP) are among the most used in their therapeutic class with a different mode of action (Srinivasan 2019; Ravele et al. 2022; Spurgeon et al. 2022; Solanki et al. 2023). They are also among the most common PCs in the aquatic environment, with concentrations ranging from 0.0001 to 15.08 µg/L (Table 1). Given that all four PCs were widely used during and after the Covid-19 pandemic (Cai et al. 2024; Fedorowski et al. 2024; Tomsone et al. 2024; Zhang et al. 2024), it is reasonable to hypothesize that their concentrations in the aquatic environment may have increased.

**Table 1:** Chemical structure, mode of action, occurrence [µg/L] of ACV, CBZ, DCF and PRP, used in the present study. N.d.: not determined in seawater

Pharmaceutical Compound	Chemical structure	Mode of action	Occurrence in seawater (µg/L; min-max values)
Acyclovir (ACV; antiviral)		It blocks virus replication by inhibiting its viral DNA polymerase, preventing viral DNA formation	n.d.
Carbamazepine (CBZ; antiepilectic)		It inactivates voltage-gated sodium channels, preventing the continuous firing of action potentials in depolarized neurons	2∙10^− 4^ – 1.41 (Bayen et al. 2013; Almeida et al. 2021)
Diclofenac (DCF; nonsteroidal anti-inflammatory)		It inhibits prostaglandin synthesis by blocking the action of cyclooxygenase (COX)-1 and − 2 enzymes	1∙10^− 4^ – 15.08 (Scheurell et al. 2009; González-Alonso et al. 2017)
Propranolol (PRP; antihypertensive)		It prevents the activity of endogenous catecholamines (i.e. epinephrine and norepinephrine at beta adrenoceptors)	5.9∙10^− 3^ – 6.33 (Capolupo et al. 2018)

Considering the lack, heterogeneity and fragmentary knowledge of ecotoxicological effects of the aforementioned PCs in marine organisms, the aim of this research was to assess the toxicity of these compounds in several key species of the first levels of the trophic chain. Thus, primary producers and primary consumers at the base of the food chain were selected, since any changes in the phyto- and zooplankton community may affect higher trophic levels, altering the marine environment. Primary producers play a key role in the marine environment, as they actively participate in nutrient cycling, and are food for filter-feeding organisms (Trenfield et al. 2015; Han et al. 2016). Even primary consumers, in particular the early life stages of crustaceans and echinoderms, are very sensitive to a wide range of contaminants. Hence, they are used in ecotoxicology. In this study, a multi-species and multi-endpoint approach, based on standardized, highly cost-effective and sensitive tests, was applied for a comprehensive assessment of the potential toxicity of different PCs. Specifically, three phytoplankton species – microalgae *Dunaliella tertiolecta*,* Phaeodactylum tricornutum*,* Skeletonema pseudocostatum* –, and two zooplankton species – crustacean *Amphibalanus amphitrite* and sea urchin *Paracentrotus lividus* – were investigated. Therefore, algal growth inhibition and chlorophyll-a content, as well as zooplankton lethal and sub-lethal endpoints – i.e. mortality, development, and behaviour – were assessed after the exposure to both environmentally relevant and high concentrations of ACV, CBZ, DCF, PRP, due to their expected increase in the aquatic ecosystem (Kock et al. 2023). By integrating chlorophyll-a inhibition for phytoplankton and development/behavioural alterations for zooplankton, we introduce a broad perspective on the non-lethal effects of PCs on primary producers and consumers.

## Materials and methods

### Pharmaceutical compounds

ACV (CAS: 59277-89-3), CBZ (CAS: 298-46-4), DCF (CAS: 15307-86-5), and PRP (CAS: 318-98-9) were purchased from Sigma Aldrich (Milano, Italy). Stock solutions of ACV, CBZ and DCF were prepared by dissolving powder in dimethyl sulfoxide (DMSO, 100%). Then, the desired test concentrations were obtained by diluting the stock solutions with filtered natural seawater (FSW, 37‰ salinity). The final DMSO concentration never exceeded 0.02% v/v. Only PRP stock solution was prepared dissolving powder in seawater. For ecotoxicological assays, the concentrations of 0.0001, 0.001 mg/L for CBZ and PRP, 0.01 mg/L for DCF were selected to evaluate their effects at environmentally relevant levels (Table 1), while 0.1, 1 and 10 mg/L - considered high concentrations compared to environmental levels – were also tested drawing on insights from previous ecotoxicological research on marine species (Aguirre-Martínez et al. 2015; Ribeiro et al. 2015; Ding et al. 2023; Nugnes et al. 2024).

### Ecotoxicological assays

#### Phytoplankton

##### Algal Growth Inhibition

The algal growth inhibition assay was carried out on the green microalga *D. tertiolecta* and on *P. tricornutum* and *S. pseudocostatum* diatoms, obtained from the CNR-IAS (National Research Council of Italy – Institute for the study of Anthropic impact and Sustainability in the marine environment, Genoa) culture collection, according to ISO 10253 (ISO 10253 2006) test method modified by using multiwell plates (Lukavský and Simmer 2001). Bioassays were performed by exposing algal suspensions (10^4^ cells/mL) to environmentally relevant (0.00001, 0.0001, 0.001, 0.01 mg/L) and high concentrations (0.1, 1, 10 mg/L) of the PCs for 72 h at 20 ± 0.5 °C with a 12:12 light: dark photoperiod and light intensity in the range of 6000–10,000 lx. All concentrations were prepared in f/2 culture medium. Three replicates were performed in 24-well plates for each concentration, also with negative (only f/2 culture medium) and solvent control (DMSO). Algal growth was blocked by Lugol’s solution after 72 h. The cells were then counted using a Bürker chamber with an inverted microscope (Leitz Diavert, Germany). The results were reported as a percentage of growth inhibition, obtained by comparing the algal growth in test solutions to the negative control.

##### Chlorophyll-a content

Chlorophyll-a was determined using the APAT CNR IRSA 9020 method (APAT-IRSA/CNR Manuali e Linee Guida 29/2003) at the end of algal growth inhibition (72 h). After exposure to different environmentally relevant concentrations (0.00001, 0.0001, 0.001, 0.01 mg/L) of each PC, thirty mL of algal species were filtered using a vacuum pump and 0.45 μm glass fiber filters. They were then extracted with 10 mL of 90% acetone at 4 °C for 24 h in the dark. Then, samples were centrifuged at 20 °C for 15 min at 3000 rpm. The supernatant was collected, while absorbance was determined by a spectrophotometer (UV-30 Scan) at 630, 647, 664, and 750 nm. Chlorophyll-a was calculated according to Broccoli et al. (2021):

Chl-a={[11.85 (Abs 664–750)-1.54 (Abs 647–750) − 0.08 (Abs 630–750)] v}/V∙L, where Abs is the absorbance measured at the specified wavelength, v is acetone extract volume (mL), V is the sample volume (L), L is the optical path (cm). The results were then reported as a percentage of chlorophyll-a inhibition, normalized to its expression in the negative control.

#### Zooplankton

##### Crustaceans

The crustacean *A. amphitrite* (II stage nauplii) was obtained from the CNR-IAS laboratory and cultured as reported by Piazza et al. (2012). The tests were carried out in multiwell plates with 10–15 organisms in 1 mL at different environmentally relevant (0.001, 0.01 mg/L) and high concentrations (0.1, 1, 10 mg/L, prepared in FSW) of PCs (or FSW for negative control and DMSO for solvent control) per well. The experiments were performed in triplicates. The plates were incubated for 48 h at 20 ± 0.5 °C in the dark. After this exposure time, two endpoints were assessed: mortality and swimming speed alteration (SSA). Dead organisms – completely motionless larvae – were counted by using a stereomicroscope. SSA was assessed by Swimming Behaviour Recorder (Faimali et al. 2006). This is a tool for recording the movement of organisms for three seconds in the dark. The percentage of SSA was calculated by comparing the average swimming speed (S) for each concentration with the negative control, as follows: SSA (%) = S (Treated − Control)/Control) × 100.

##### Echinoderms

Adults of the sea urchin *P. lividus* were sampled in the Ligurian Sea and brought to the CNR-IAS laboratory in a refrigerated bag according to Amemiya (1996). An injection of KCl (0.5 M) was administered into the coelomic cavity to induce spawning (Morgana et al. 2016). Spermatozoa and eggs were taken from three male and three female organisms, as reported by Morroni et al. (2016). Sperms were sampled from the genital pores and stored dry in a sterile tube at 4 °C before their use. Eggs were collected in FSW at room temperature. The eggs and sperms of three different organisms were mixed. Then, the egg suspension of 1000 eggs/mL was combined with 10 µl of sperm diluted 1:1000 in FSW, and incubated at 18 ± 1 °C.

After 40 min, fertilization was checked under a stereomicroscope (Zeiss, Germany), by establishing the presence of a fertilization membrane. Acceptability criteria were fixed at > 70% fertilization rate, according to Beiras et al. (2003). Then, embryo toxicity tests were carried out in six multiwell plates by mixing 1 mL of zygote solution and 9 mL of PC solution (0.001, 0.01, 0.1, 1, 10 mg/L, prepared in FSW), or FSW/DMSO for controls in each well, in triplicates. The plates were incubated for 72 h at 18 ± 1 °C in the dark (Sartori et al. 2017). After 72 h, 100 pluteus larvae were fixed in 2% paraformaldehyde (PAF) in FSW and each replicate was counted using a stereomicroscope. Depending on their morphology and synchronous development, they were classified as ‘developed’ and ‘not-developed’ (Gambardella et al. 2013). Before fixation, plutei were used to evaluate SSA by SBR. Swimming speed was recorded for 5 s in multiwell plates with 20–25 organisms and 1 mL of PC environmentally relevant (0.001, 0.01) and high concentrations (0.1, 1, 10 mg/L, prepared in FSW) per well, in four replicates. SSA (%) was calculated as described above for barnacle nauplii.

### Statistical analysis

Data of ecotoxicological bioassays are reported as mean ± SE (standard error) from three independent tests. LC50 (concentration of PCs inducing mortality in 50% of exposed organisms after 48 h) and EC50 (concentration of PCs causing SSA or algal growth inhibition or developmental anomalies in 50% of exposed organisms after 48–72 h) and Confidence Limits (95%) were estimated with Spearman–Karber analysis (Finney 1978). ANOVA (One-way analysis of variance) and Dunnett’s multiple comparison tests were used to determine the statistical significance from negative control (*p *<* 0.05, **p *<* 0.001 and ***p *<* 0.0001), estimating NOEC (No Observed Effect Concentration) and LOEC (Lowest Observed Effect Concentration) values, by using Graphpad prism 5 software. These statistical approaches ensured that reported significance levels (p-values) accurately reflected variability within and across experimental groups.

### Risk assessment

The environmental risk of the selected PCs was assessed as described by the European Medicine Agency (EMA 2006) and Technical Guidance Document on Risk Assessment. Part 2. (TGD; EC 1996^2003^) and as previously reported for other PCs (Aguirre-Mart﻿ínez et al. 2015; Cortez et al. 2019). Risk Quotient (RQ) values were calculated according to the literature, by dividing the Measured Environmental Concentration (MEC) in seawater by Predicted No-Effect Concentration (PNEC) values, as measured in this study. PNEC was estimated by the ratio between LC50/EC50 and an Assessment Factor (AF; Table S1) of 1000, according to the ecotoxicological tests performed in this study. To represent the worst-case scenario, the highest seawater MEC value reported in the literature for CBZ, DCF and PRP was used (Table 1). To the best of our knowledge, there are no studies reporting MEC values of ACV in seawater. Therefore, the highest MEC value reported in freshwater (Nugnes et al. 2024) was used. When LC50/EC50 values could not be calculated, the highest tested concentration (10 mg/L) was used, according to Aguirre-Martinez and colleagues (2015). For the purpose of environmental risk interpretation, the RQ value < 0.1 indicates no risk, 0.1 ≤ RQ < 1 suggests a medium risk, and RQ ≥ 1 means high risk (Yao et al. 2017).

## Results

### Phytoplankton

**Fig. 1:**
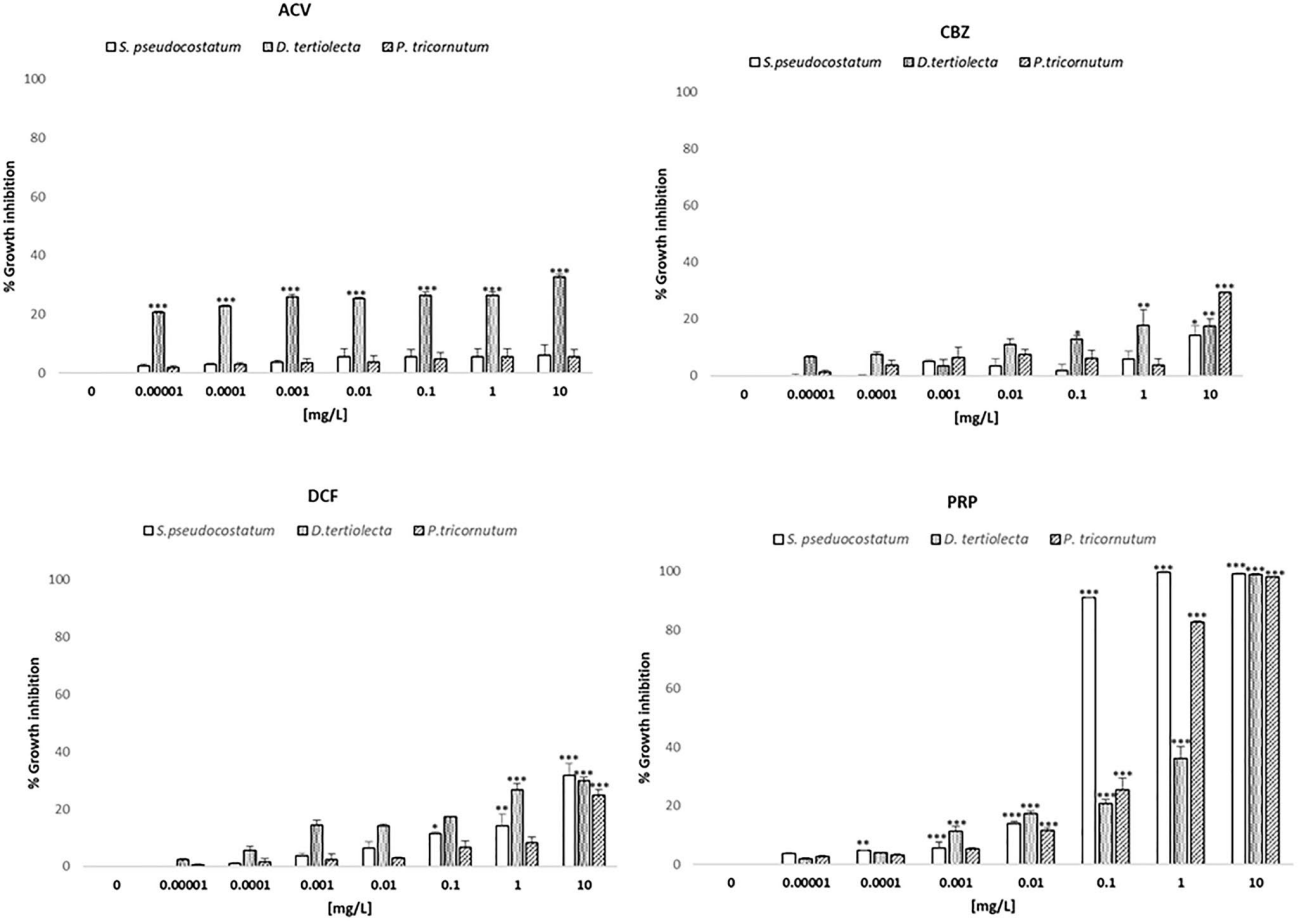
Growth inhibition in phytoplankton species after exposure to different concentrations (0.00001, 0.0001, 0.001, 0.01, 0.1, 1, 10 mg/L) of the four PCs (Acyclovir - ACV, Carbamazepine – CBZ, Diclofenac - DCF and Propranolol - PRP) and to the negative control (0 mg/L), corresponding to filtered natural seawater. Data represent mean ± standard error of the 3 replicates. *p *<* 0.05, **p *<* 0.001 and ***p *<* 0.0001 compared to negative control (One way ANOVA, Dunnett’s multiple comparison test)

The algal growth inhibition obtained in phytoplankton species after 72 h of exposure to different PC concentrations is reported in Fig. 1.

No significant algal growth inhibition was observed in solvent control (< 10% effect). All investigated PCs impaired the growth of the three algal species in a dose-dependent way. Among the compounds, PRP caused the greatest ecotoxicological effect, reporting an EC50 toxicity (from 0.03 to 1.14 mg/L; Table 2), while all other PCs induced < 40% effects (EC50 > 10 mg/L).

**Table 2:** LC50 and EC50 values with confidence limits (95%), expressed in mg/L, calculated for phyto- and zooplankton after exposure to pharmaceutical compounds

		Exposure time	Endpoint	ACV	CBZ	DCF	PRP
Phytoplankton	*D.tertiolecta*	72 h	Growth inhibition	> 10	> 10	> 10	1.14 (0.25–5.14)
	*P.tricornutum*	72 h	Growth inhibition	> 10	> 10	> 10	0.25 (0.14–0.43)
	*S.pseudocostatum*	72 h	Growth inhibition	> 10	> 10	> 10	0.03 (0.02–0.04)
Zooplankton	*A.amphitrite*	48 h	Mortality Behaviour	> 10 > 10	> 10 > 10	> 10 > 10	0.41 (0.35–0.49) 0.14 (0.10–0.21)
	*P.lividus*	72 h	Larval development Behaviour	> 10 > 10	> 10 > 10	> 10 > 10	0.21 (0.18–0.26) 0.17 (0.13–0.21)

Regarding ACV, a significant growth inhibition was only observed in *D. tertiolecta*, starting at the lowest concentration (0.00001 mg/L). The highest CBZ concentration (10 mg/L) significantly affected the growth of all species; the green alga *D. tertiolecta* was significantly inhibited even at lower concentrations (0.1 mg/L, 1 mg/L), unlike diatoms. Regarding DCF, the highest effect (30%) was observed at 10 mg/L for all algae. Among them, *S. pseudocostatum* and *D. tertiolecta* were the most sensitive species to this PC, being significantly affected from 0.1 mg/L and 1 mg/L, respectively. The same sensitivity among species was also observed after exposure to PRP, where *S. pseudocostatum* was sensitive to almost all concentrations, followed by *D. tertiolecta* and *P. tricornutum.*

**Fig. 2:**
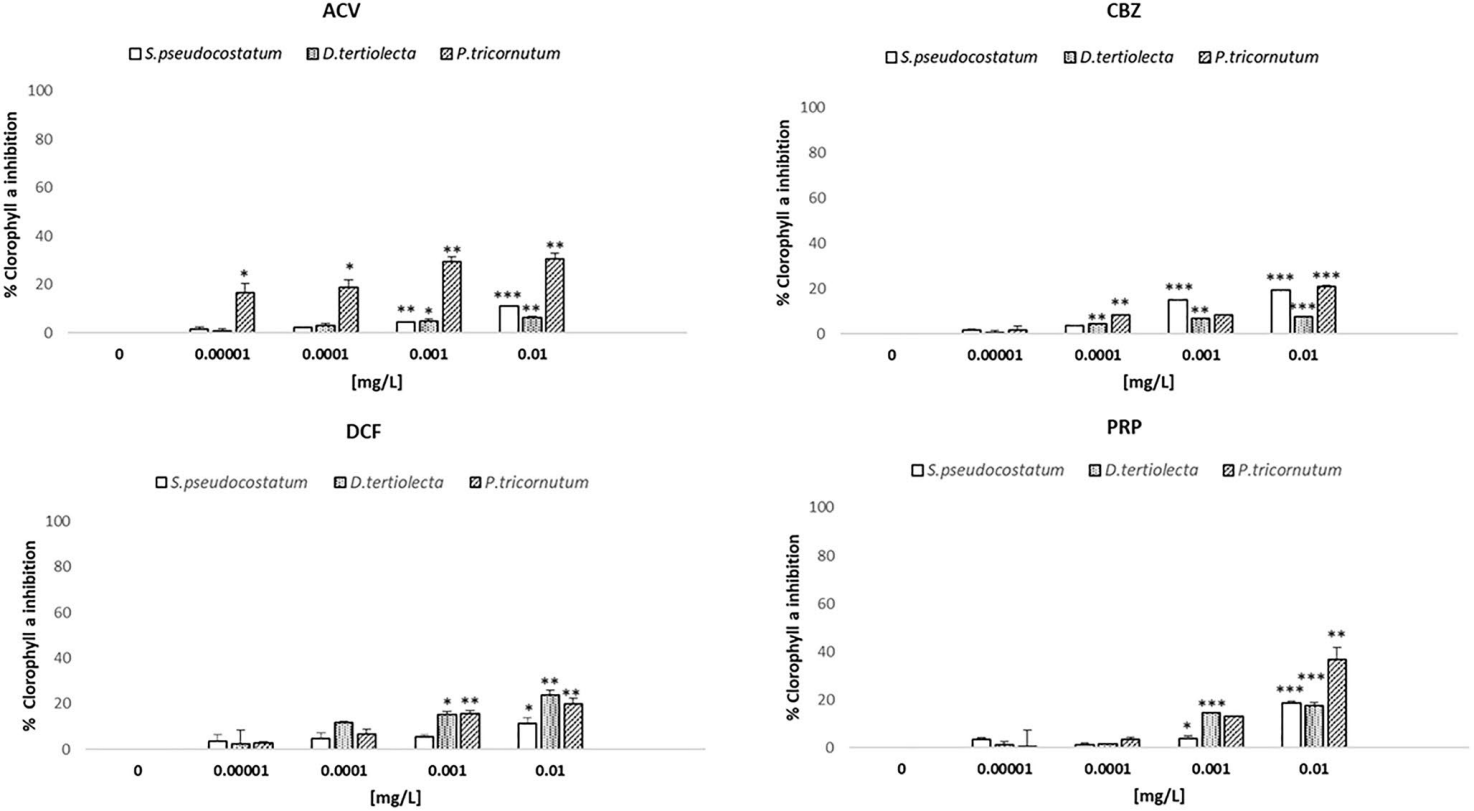
Chlorophyll-a in phytoplankton species after exposure to different concentrations (0.0001, 0.001, 0.01 mg/L) of the four PCs (Acyclovir - ACV, Carbamazepine – CBZ, Diclofenac - DCF and Propranolol - PRP) and to the negative control (0 mg/L), corresponding to filtered natural seawater. Data represent mean ± standard error of the 3 replicates. *p *<* 0.05, **p *<* 0.001 and ***p *<* 0.0001 compared to negative control (One way ANOVA, Dunnett’s multiple comparison test)

The chlorophyll-a inhibition obtained in phytoplankton species after 72 h exposure to the four PCs is reported in Fig. 2.

Considering that chlorophyll-a inhibition is a sub-lethal endpoint, it should not be determined at concentrations higher than 0.01 mg/L due to growth inhibition greater than 30%. All PCs reduced the amount of chlorophyll-a in a dose-dependent manner. Among the selected PCs, ACV and CBZ significantly inhibited chlorophyll-a already at the lowest concentrations (0.00001 and 0.0001 mg/L; Table 3). Conversely, significant DCF and PRP effects were observed from 0.001 mg/L.

**Table 3:** Risk quotient values of ACV, CBZ, DCF and PRP calculated considering LC50 (mortality) and EC50 (growth inhibition, behaviour and larval development) values for acute toxicity assays of phyto (*D. tertiolecta*,* P. tricornutum*,* S. pseudocostatum*)- and zooplankton (*A. amphitrite*,* P. lividus*)

		Phytoplankton			Zooplankton			
		***D. tertiolecta***	***P. tricornutum***	***S. pseudocostatum***	***A. amphitrite***		***P. lividus***	
			**Growth inhibition**		**Mortality**	**Behaviour**	**Larval development**	**Behaviour**
RQ	**ACV**	0.159	0.159	0.159	0.159	0.159	0.159	0.159
	**CBZ**	0.141	0.141	0.141	0.141	0.141	0.141	0.141
	**DCF**	**1.5**	**1.5**	**1.5**	**1.5**	**1.5**	**1.5**	**1.5**
	**PRP**	**5.55**	**25.32**	**211**	**15.44**	**45.21**	**30.14**	**37.23**

Bold values indicate a high environmental risk

### Zooplankton

**Fig. 3:**
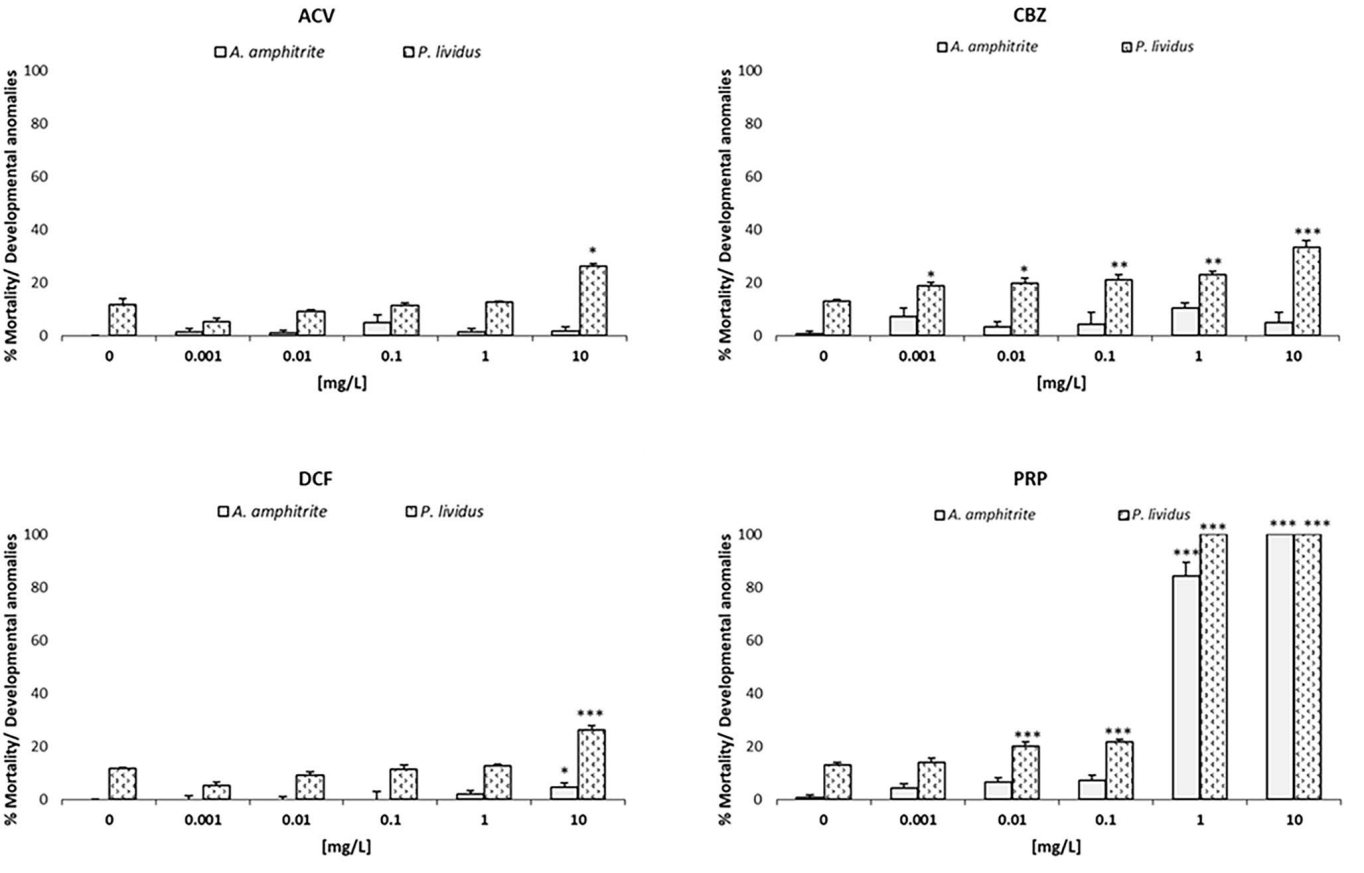
Mortality and developmental anomalies in zooplankton species after exposure to different concentrations (0.001, 0.01, 0.1, 1, 10 mg/L) of the four PCs (Acyclovir - ACV, Carbamazepine – CBZ, Diclofenac - DCF and Propranolol - PRP) and to the negative control (0 mg/L), corresponding to filtered natural seawater. Data represent mean ± standard error of the 3 replicates. *p *<* 0.05, **p *<* 0.001 and ***p *<* 0.0001 compared to negative control (One way ANOVA, Dunnett’s multiple comparison test)

The ecotoxicological effects on zooplankton after 48 and 72 h exposure to different PCs are reported in Fig. 3.

No effects were recorded in barnacle and sea urchin larvae after their exposure to the solvent control (< 10% effect). Among PCs, PRP caused the greatest effect in crustaceans and echinoderms, reporting a toxicity index of LC50 = 0.41 (0.35–0.49) mg/L for *A. amphitrite*; EC50 = 0.21 (0.18–0.26) mg/L for *P. lividus;* Table 2), while all other PCs induced < 40% effects.

No significant effects in terms of mortality were observed in *A. amphitrite* nauplii (< 10%) exposed to both ACV and CBZ. Conversely, percentages of significant developmental anomalies were observed in the sea urchin *P. lividus* at the highest ACV concentration (10 mg/L) and from 0.001 mg/L CBZ. Regarding DCF, a toxic effect was observed only at 10 mg/L for both species, showing a higher sensitivity in sea urchin development compared to barnacle mortality. *A. amphitrite* survival was significantly affected at 1 mg/L and 10 mg/L of the antihypertensive PC (PRP). Conversely, the development of *P. lividus* was significantly impaired from 0.01 mg/L. Only the highest concentration (10 mg/L) caused 100% mortality and developmental anomalies in nauplii and plutei, respectively.

**Fig. 4:**
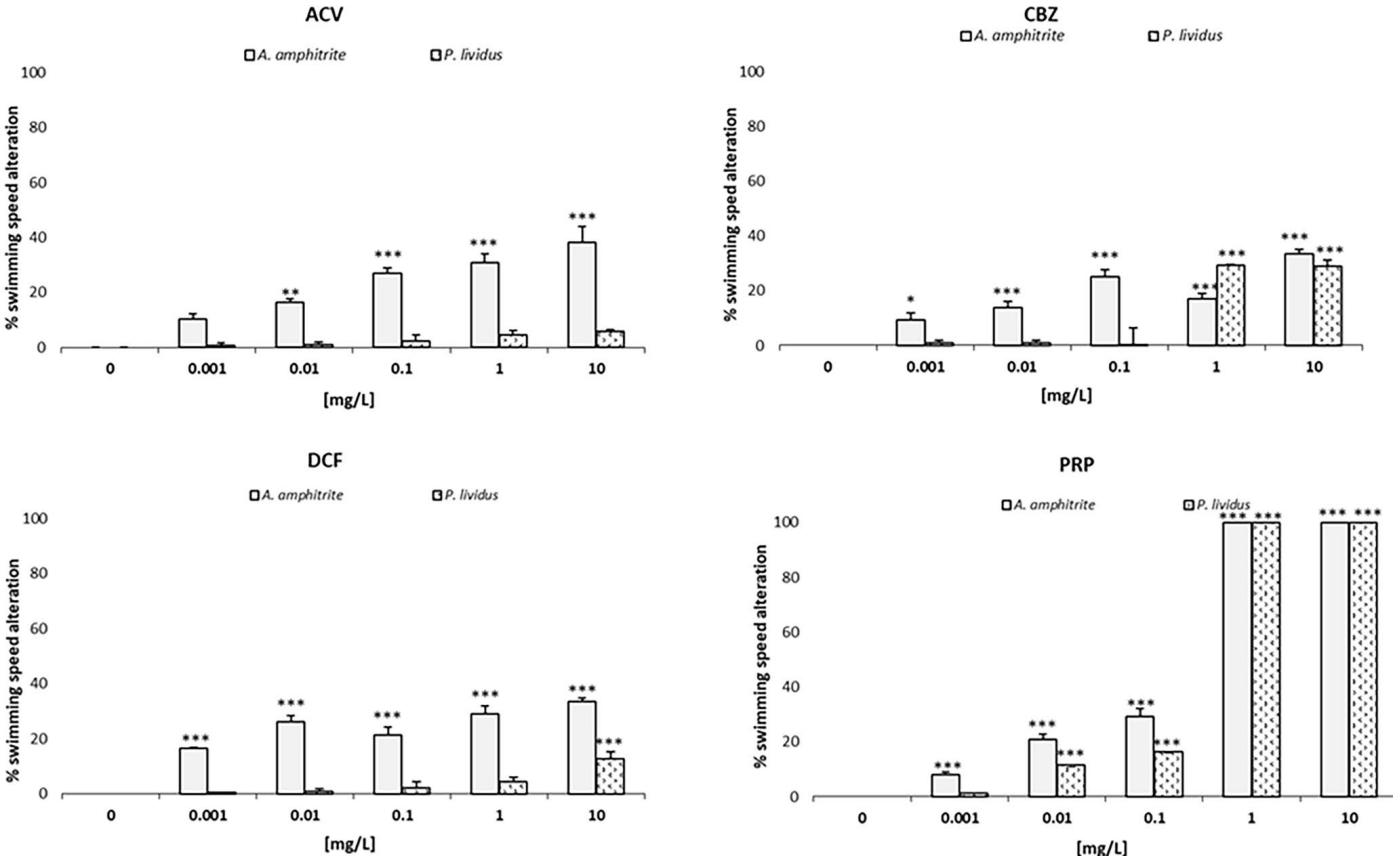
Swimming speed alteration (SSA) in zooplankton species after exposure to different concentrations (0.001, 0.01, 0.1, 1, 10 mg/L) of the four PCs (Acyclovir - ACV, Carbamazepine – CBZ, Diclofenac - DCF and Propranolol - PRP) and to the negative control (0 mg/L), corresponding to filtered natural seawater. Data represent mean ± standard error of the 3 replicates. *p *<* 0.05, **p *<* 0.001 and ***p *<* 0.0001 compared to negative control (One way ANOVA, Dunnett’s multiple comparison test)

The swimming speed alteration (SSA) measured in zooplankton species after 48 and 72 h of PCs exposure is reported in Fig. 4.

The most significant behavioural impairment was caused by PRP, reporting EC50 values (0.14 (0.10–0.21) mg/L for *A. amphitrite*; 0.17 (0.13–0.21) mg/L for *P. lividus;* Table 2), while all other PCs induced < 40% SSA (EC50 > 10 mg/L; Table 2). Indeed, significant SSA inhibition was observed from 0.01 mg/L of ACV in *A. amphitrite*. Conversely, *P. lividus* did not show any toxicity (< 10%). CBZ and DCF caused a 33% reduction in naupliar behaviour only at the highest concentration (10 mg/L); nevertheless, a significant impairment was observed starting from the lowest concentration of both compounds (0.001 mg/L). Regarding *P. lividus*, a significant behavioural alteration was recorded starting from 1 mg/L for CBZ and 10 mg/L for DCF. Similarly to CBZ and DCF, the lowest concentration (0.001 mg/L) of the antihypertensive PRP significantly reduced swimming speed in *A. amphitrite*. Conversely, *P. lividus* showed significant behavioural alterations from 0.01 mg/L (Table S1).

Regarding zooplankton, SSA in barnacles was recorded at lower concentrations than mortality, while SSA in sea urchins occurred at higher concentrations than larval development anomalies (Table 3). These findings showed a difference in sensitivity of the two endpoints depending on tested species. Considering the most sensitive endpoint of each species, significant behavioural alterations of *A. amphitrite* were recorded at concentrations ranging from 0.001 to 0.01 mg/L; conversely, significant developmental anomalies in *P. lividus* were reported between 0.01 and 10 mg/L for almost all PCs.

The risk of selected PCs in the marine ecosystem can be quantified by RQ values (Table 3). For ACV, CBZ and DCF it was not possible to determine LC50/EC50 values for any assays, hence, the highest tested concentration (10 mg/L) was used. By analyzing all species and endpoints, ACV and CBZ pose a medium risk to marine organisms (0.1 ≤ RQ < 1), while a high risk was calculated for DCF and PRP (RQ ≥ 1). RQ values were higher in PRP than in DCF.

## Discussions

In this study, we investigated the toxicity of four PCs in marine phyto- and zooplankton, through environmentally relevant and high exposures. A multi-species and multi-endpoint approach was adopted to provide a more comprehensive risk assessment of PCs in the marine ecosystem. Environmentally relevant concentrations of these PCs belonging to different therapeutic classes do not induce lethal and sub-lethal responses in either phyto- and zooplankton. However, these PCs may still be potentially toxic in prolonged exposure or in mixture among them or with other contaminants, highlighting the importance of further performing chronic exposure or PC combined exposure to verify this hypothesis.

Our findings on CBZ, DCF and PRP confirm the literature available so far on the first levels of the marine trophic chain, showing no effects on algal growth inhibition (De Lorenzo & Fleming. 2008; Claessens et al. 2013; Tsiaka et al. 2013; Duarte et al. 2020; Ding et al. 2023) and on zooplankton (crustacean, echinoderm) survival, development and reproduction at environmental levels (Karaaslan and Parlak et al. 2016; Barrick et al. 2022; Prato et al. 2023).

Growth inhibition is the traditional endpoint for evaluating the toxicity of contaminants in algae, as indicated by international guidelines (i.e. UNI EN ISO 10253/2016, ISO 8692/2012). In this study, we proposed chlorophyll-a inhibition as a useful parameter for studying PC effects at environmentally relevant concentrations. This parameter is considered an indicator of algal photosynthetic activity (Tretiach et al. 2007; Zhang et al. 2012; Xin et al. 2021) which has been recently reported to be sensitive to PCs (Mojiri et al. 2022). In our study, it was not possible to calculate EC50 values for PCs at environmentally relevant concentrations. High PCs concentrations were not included in this study for chlorophyll-a analysis since they were toxic to marine algae (Fig. 1), probably due to cell structures and organelle damage ascribed to ROS homeostasis disturbance (Xiong et al. 2019). However, chlorophyll-a inhibition was more sensitive than growth inhibition, as reported from LOEC values (Table S2). Accordingly, scientific reports suggest that PCs can affect algal biochemical parameters, also in terms of chlorophyll or carotenoid alteration (Lushchak 2011). Anti-inflammatory, antihypertensive, and antiepileptic compounds have significantly reduced chlorophyll content in several freshwater algal species. For instance, Wang et al. (2020) observed a significant chlorophyll content inhibition due to DCF in the green algae *Scenedesmus obliquus*. Similar results were also observed in other species (i.e. *S. obliquus*,* Chlorella pyrenoidosa* and *Chlorella vulgaris)* after exposure to CBZ and PRP (Lodowska et al. 2003; Zhang et al. 2012). Our results are in line with these studies, confirming that chlorophyll content can be significantly reduced by several therapeutic classes of PCs even in marine species, from green algae to diatoms. Regarding zooplankton, we analysed both traditional and innovative endpoints. Traditional responses – including mortality, development – in ecotoxicology are typically used for assessment or regulatory purposes (Bertram et al. 2024), following standardized protocols (i.e. ISO, ASTM). Although the innovative responses (i.e. behaviour) are not regulatory endpoints, they have been proposed as a broad indicator of sub-lethal toxicity in aquatic ecotoxicology (Gravato et al. 2014), since they are sensitive to pollutants’ exposure, directly or indirectly linked to the fitness of animals, and represent an integrated response at the organism level (Faimali et al. 2017). In this study, we demonstrated the swimming behaviour sensitivity towards PRP, as representative of antihypertensive PCs. Although swimming was not more affected than regulatory endpoints in marine zooplankton exposed to other PCs, we recommend a multi-endpoint approach based on both traditional and behavioural responses to evaluate the potential risks of PCs in the marine environment, since any altered responses may be associated with reduced fitness and survival, with consequences at population level (Bridges 1997).

The antiviral ACV has been detected in surface waters (i.e. wastewater treatment plants, rivers; Azuma et al. 2016; Funke et al. 2016). However, its effects have not been investigated yet in any marine species. With this paper, we contribute to filling the gap, for the first time providing new insights into its toxicity for marine biota. The ecotoxicological effects of ACV in the freshwater ecosystem have recently drawn attention. Here, chronic toxic effects on green algae growth (Almeida et al. 2021), and on crustacean reproduction were observed (Nugnes et al. 2024) at concentrations lower than 10 mg/L. Although we did not find toxicity in marine algae and crustaceans up to 10 mg/L, algal growth and zooplankton behaviour/development were significantly affected, being responsible for a medium risk assessment. The latter was calculated adopting the freshwater MEC instead of the seawater-specific one, due to the lack of seawater data. This may introduce some uncertainty in risk estimation, as environmental factors (i.e. salinity and organic matter content) can influence the fate, bioavailability, and toxicity of ACV in marine ecosystems (Hall et al.^ 2008^). To enhance the accuracy of risk assessments, future studies should focus on determining seawater-specific MEC values and evaluating their ecological implications, ensuring a more reliable estimation of potential environmental risks. In any case, as suggested by our data, even though it may impair the first levels of the marine trophic chain at higher concentrations than freshwater ones, this antiviral PC is confirmed to be a hazardous compound for the aquatic ecosystem, as recently reported for freshwater ecosystem (Nugnes et al. 2024).

Phyto- and zooplankton growth and survival were not affected by CBZ exposure, unlike other endpoints – i.e. chlorophyll inhibition, behaviour, and development – thus confirming data available in the literature on marine microalgae and zooplankton (Almeida et al. 2014; Aguirre-Martínez et al. 2015; Prato et al. 2023). Regarding sea urchins, we report a significant effect – close to 40% – on the larval development, which is sensitive to short-term exposure of CBZ, as demonstrated in marine zooplankton larvae, including sea urchins and mussels (Aguirre-Martínez et al. 2015; Franzellitti et al. 2019). Thus, CBZ may interfere with multiple processes in early zooplankton development, modulating the uptake and release of neurotransmitters – including gamma-aminobutyric acid (GABA) (Franzellitti et al. 2019; Mezzelani et al. 2021). Sea urchins have a GABAergic system that can be affected by PCs (Fogliano et al. 2024). CBZ may impact on such system, by altering their early development and swimming behaviour, since the latter depends on the GABAergic system (Katow et al. 2013). These findings may also explain swimming behaviour impairment in barnacle nauplii at all CBZ concentrations. Similar to sea urchins, barnacles have a GABAergic system that may affect motility (Gallus et al. 2009). These findings on two zooplankton species taken together suggest that CBZ can impair development and swimming in the early stages.

For all endpoints and species, a medium risk of 0.141 was estimated for CBZ. The presence of CBZ has been defined unsafe for the marine ecosystem, due to high RQ values (> 1; Almeida et al. 2014).

Exposure to DCF did not cause toxic effects on marine phyto- and zooplankton in terms of EC50s. In all species we observed a significant reduction in algal growth, confirming the study of Ding et al. (2023), who reported growth inhibition in *P. tricornutum* and *Euglena* sp. only at high concentrations. We found similar results in zooplankton, where DCF affected *P. lividus* development only at concentrations exceeding environmentally relevant concentrations, as previously reported for another sea urchin species (*Arbacia lixula)*, where development impairment occurred at 50 mg/L. In addition, the swimming behaviour of both zooplankton species was affected. While only high DCF levels (10 mg/L) altered echinoderm swimming speed, in barnacle nauplii swimming was affected at all concentrations. DCF has been reported to reduce swimming activity and exploration ability in several freshwater organisms, including crustaceans (Melvin 2016; Peltzer et al. 2019; Di Cicco et al. 2021). This anti-inflammatory PC may alter mechanoreception in freshwater and marine organisms, by inducing neurotoxic effects, and inhibiting neurotransmission (i.e. cholinesterase activity; Bebianno & Gonzalez-Rey 2015; de Oliveira et al. 2016). The nervous system in early-stage barnacles is largely cholinergic (Falugi 1988), therefore DCF exposure may alter it, causing significant changes in behaviour, as observed in this study.

All ecotoxicological responses measured in phytoplankton and zooplankton pointed to a high environmental risk (RQ > 1) for DCF. These data confirm previous literature reports on freshwater species (algae, crustaceans, fish), reporting similar RQ values (1.5–3.5; Korkmaz et al. 2022; Szymczycha et al. 2020).

Among PCs, PRP caused the most toxic effects in all phytoplankton and zooplankton species, thus posing a high environmental risk. Regarding primary producers, all microalgae species were significantly affected in terms of growth inhibition. These findings confirm similar results reported in the literature for the same marine diatoms (*P. tricornutum*,* S. pseudocostatum*) and other green microalgae (*Desmodesmus subspicatus;* Claessens et al. 2013; Cleuvers 2003; Petersen et al. 2014). Using a multi-species approach, this study determined a range of different sensitivity in marine algae exposed to PRP. Thus, *S. pseudocostatum* was the most sensitive species, followed by *P. tricornutum* and *D. tertiolecta* (Table 2). These findings suggest that marine diatoms (*S. pseudocostatum*,* P. tricornutum)*, rather than green algae, are the most affected by PRP exposure. The antihypertensive PRP exerts its action by blocking β-adrenergic receptors not present in algae and plants (Duarte et al. 2020). However, some diatoms have ‘plant receptor-like kinases’ mainly responsible for maintaining oxidative balance and the production of ROS (Galindo-Trigo et al. 2016). These receptors have biochemical properties similar to β-adrenergic receptors, making up a likely target for PRP (Duarte et al. 2020). Although it is not yet completely understood how PRP can interfere with algal growth, it could block such receptors in marine diatoms, thus explaining their higher sensitivity than green algae to this PC in terms of EC50 and RQ values. When algal species lack these receptors, high levels of ROS are observed, resulting in photosynthetic inefficiency and increased cell death (Burdiak et al. 2015). Further investigations linked to ROS production in both marine diatoms and green algae are needed to verify this assumption and confirm the high sensitivity of diatoms to PRP exposure. Different from other PCs, PRP caused a toxic effect even in zooplankton species. Thus, lethal and sub-lethal effects were measured in both barnacle nauplii and echinoderm larvae in the same order of magnitude, but still at higher concentrations than those detected in the aquatic environment. Our results are in line with Karaaslan and Parlak (2016), reporting similar values (EC50: 0.23 mg/L) based on developmental anomalies occurring in *P. lividus* exposed to PRP for 72 h. Through its β-blocking action, high PRP levels (i.e. 12.5 mg/L) arrested the development in the first early stages (embryos, larvae) of aquatic organisms (Ribeiro et al. 2015; Capolupo et al. 2018), even if significant developmental anomalies had already occurred at lower concentrations (0.0005 mg/L, Capolupo et al. 2018). Our findings confirm literature data on the effects of PRP in impairing sea urchin development, besides proving new ecotoxicological effects, including effects on behaviour, ascribed to PRP. Since sea urchins are characterized by β- adrenergic receptors, PRP may exert its ecotoxicological effects by interfering with these targets (Capolupo et al. 2018). Moreover, for the first time, we reported PRP ecotoxicological effects on another zooplankton organism: the crustacean *A. amphitrite*. Although no studies are available on PRP toxicity in barnacles, it has been shown that exposure to the antihypertensive atenolol inhibited *A. amphitrite* settlement (Al-Aidaroos et al. 2017), thus demonstrating the sensitivity of this barnacle species to β-blocker compounds. Generally, β-blockers can act on the nervous system by blocking neurotransmitter activities (Frishman 2003). Among them, catecholamines are involved in metabolic processes of marine invertebrate larvae (Croll et al. 1997; Wang et al. 2006), such as barnacle metamorphosis (Al-Aidaroos et al. 2017), cellular differentiation, and morphogenesis in the early developmental stages of echinoderms (Anitole-Misleh & Brown 2004). Although catecholamine involvement has not yet been documented for the survival or swimming of barnacle and echinoderm larvae, the findings could suggest a potential mode of action of antihypertensive β-blockers in the selected zooplankton species. A high environmental risk (RQ > 1) of PRP was found in this study by analyzing all species and endpoints. The estimated RQ values, ranging from 5 to 211, are in line with Capolupo et al. (2018), reporting a severe risk (RQ = 17.29) for *P. lividus* exposed to PRP.

To the best of our knowledge, this study represents the first attempt to assess the environmental risk of PCs representative of antiviral, antihypertensive, anticonvulsants and anti-inflammatory compounds, through RQ from measured ecotoxicological responses across multiple marine species. Our findings indicate that all PCs pose a risk to the marine environment – from moderate to high - taking into account both lethal and sub-lethal effects on the marine producers and consumers. For RQ calculation, it is important to acknowledge the limitations associated of using the highest tested concentration (10 mg/L) when LC50/EC50 values could not be determined. Although this approach follows established methodologies (Aguirre-Mart﻿ínez et al. 2015) and ensures a precautionary assessment, it may lead to an overestimation of the environmental risk, particularly for compounds like ACV and CBZ, for which no clear toxicity thresholds were identified in this research. This limitation highlights the need for further studies using more sensitive endpoints to refine risk estimates and better estimate potential environmental effects.

## Conclusions

This study provides new insights into the ecotoxicological effects of four PCs on marine organisms belonging to different levels of the trophic chain. With our multi-species and multi-endpoint approach it was possible to assess the toxicity of these emerging contaminants, estimating environmental risks, ranging from medium to high. This study suggests that all the selected PCs may pose a risk to marine phyto- and zooplankton. Therefore, there is an urgent need to monitor their presence and effects in the marine environment, by including these PCs in monitoring programs, before their potential transfer along the trophic chain. Future research should focus on chronic toxicity studies to assess long-term effects at environmentally relevant concentrations. Since PCs in the aquatic environment undergo both abiotic and biotic transformations, often generating persistent and biologically active metabolites (Han & Lee 2017), their potential effects on non-target marine organisms should also be addressed. Additionally, investigating the combined effects of pharmaceutical mixtures is essential to better understand potential synergistic or antagonistic interactions. Further studies on molecular and biochemical responses in marine organisms would clarify the mechanisms involved in the observed toxicity.

## Supplementary Information

Below is the link to the electronic supplementary material.

Supplementary Material 1

## Data Availability

No datasets were generated or analysed during the current study.
