# Supplementary material for: Pharmaceutical compounds in marine ecosystems: ecotoxicological effects and risk assessment in phytoplankton and zooplankton
Source: Ecotoxicology. 2025 Sep 12;34(9):1869–83. doi: 10.1007/s10646-025-02960-2 (PMC12553569; doi:10.1007/s10646-025-02960-2)
Supplement: Supplementary file 1 — Supplementary Material 1 [file 10646_2025_2960_MOESM1_ESM.docx]

**Nugnes et al.**

**Supplementary tables**

**Table S1** Assessment factors proposed for deriving PNECwater for saltwater for different data sets (TGD, 2003).

| **Data set** | **Assessment Factor** |
| --- | --- |
| Lowest short-term L(E)C50 from freshwater or saltwater representatives of three taxonomic groups (algae, crustaceans and fish) of three trophic levels | 10000 |
| Lowest short-term L(E)C50 from freshwater or saltwater representatives of three taxonomic groups (algae, crustaceans and fish) of three trophic levels, + two additional marine taxonomic groups (e.g. echinoderms, molluscs) | 1000 |
| One long-term NOEC (from freshwater or saltwater crustacean reproduction or fish growth studies) | 1000 |
| Two long-term NOECs from freshwater or saltwater species representing two trophic levels (algae and/or crustaceans and/or fish) | 500 |
| Lowest long-term NOECs from three freshwater or saltwater species (normally algae and/or crustaceans and/or fish) representing three trophic levels | 100 |
| Two long-term NOECs from freshwater or saltwater species representing two trophic levels (algae and/or crustaceans and/or fish) + one long-term NOEC from an additional marine taxonomic group (e.g. echinoderms, molluscs) | 50 |
| Lowest long-term NOECs from three freshwater or saltwater species (normally algae and/or crustaceans and/or fish) representing three trophic levels + two long-term NOECs from additional marine taxonomic groups (e.g. echinoderms, molluscs) | 10 |

**Table S2** LOEC values (mg/L) calculated using Dunnett’s multiple comparison test (One way ANOVA).

|  | LOEC | | | | |
| --- | --- | --- | --- | --- | --- |
|  | **Endpoint** | **ACV** | **CBZ** | **DCF** | **PRP** |
| *D.tertiolecta* | Growth inhibition | 0.00001 | **0.1** | 1 | **0.001** |
|  | Chlorophyll-a inhibition | 0.001 | **0.0001** | **0.001** | **0.001** |
| *P.tricornutum* | Growth inhibition | >10 | 10 | 10 | **0.01** |
|  | Chlorophyll-a inhibition | 0.00001 | **0.0001** | **0.001** | **0.01** |
| *S.pseudocostatum* | Growth inhibition | >10 | 10 | 0.1 | **0.0001** |
|  | Chlorophyll-a inhibition | 0.001 | **0.001** | **0.01** | **0.001** |
| *A.amphitrite* | Mortality | >10 | >10 | 10 | 1 |
|  | Behaviour | 0.01 | **0.001** | **0.001** | **0.001** |
| *P.lividus* | Larval development | 10 | **0.001** | 10 | **0.01** |
|  | Behaviour | >10 | 1 | >10 | 1 |
